# Supplementary material for: Analysis of the demand for gastronomic tourism in Andalusia (Spain)
Source: PLoS One. 2021 Feb 5;16(2):e0246377. doi: 10.1371/journal.pone.0246377 (PMC7864416; doi:10.1371/journal.pone.0246377)
Supplement: S1 Survey — (DOC) [file pone.0246377.s001.doc]

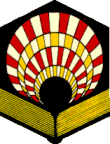
[
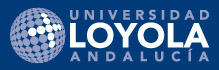
](http://www.uloyola.es/)

**SURVEY: ANALYSIS OF THE DEMAND FOR GASTRONOMIC TOURISM IN ANDALUSIA (SPAIN)**

Survey for the analysis of gastronomic tourism demand in the Andalusia, Spain

**Protocol**

We are conducting a research study on the social reality of gastronomic tourism in Andalusia. We kindly request your collaboration in this rigorous scientific study on the demand of gastronomic tourism in Andalusia.

Thank you for agreeing to take part in this ambitious project that aims to publicize the gastronomy. In order for the information from this research to be the most helpful, it is important that you try to be as accurate, complete, and honest as possible with your answers.

The information you provide in the questionnaire will be kept in the strictest confidentiality. Once we have analyzed the data, a report will be delivered to the Tourism Delegation of Cordoba for dissemination to all the companies in the sector that have participated in the survey or will be published in a international journal.

Thank you for your participation

1. **PERSONAL CHARACTERISTICS OF GASTRONOMIC TOURISTS**
2. Gender:

**1** ❑ Male

**2** ❑ Female

1. Age:

**1** ❑ 18-29 years old

**3** ❑ 30-39 years old

**4** ❑ 40-49 years old

**5** ❑ 50-59 years old

**6** ❑ More than 60 years old

1. Education level

**1** ❑ No studies completed

**2** ❑ Primary studies

**3** ❑ Secondary studies

**4** ❑ Higher studies

1. Marital status

**1** ❑ Single

**2** ❑ Married

**3** ❑ Divorced/separated

**4** ❑ Other status

1. Level of monthly income of the family unit

**1** ❑ + than 2500 euros

**2** ❑ de 2.001 a 2.500 euros

**3** ❑ de 1.501 a 2.000 euros

**4** ❑ 1.001 a 1.500 euros

**5** ❑ Less than 1000 euros

1. Whom did you travel with?

**1** ❑ Alone

**2** ❑ Accompanied by my partner

**3** ❑ With family members

**4**  ❑ With friends

1. Where are you from?

**1** ❑ Andalusia

**2** ❑ Rest of Spain (except Andalusia)

**3** ❑ European Union (except Spain)

**4**  ❑ Rest of the world (except European Union)

1. Employment status

**1** ❑ Employed by others

**2** ❑ Self-employed

**3**  ❑ Retired

**4** ❑ Unemployed

**5**  ❑ Student

1. Duration of the trip

**1** ❑ Menos de 24 horas Less than 24 hours

**2**  ❑ 2-3 days

**3**  ❑ More than 3 days

1. Daily expenditure

**1** ❑ Less than 30 euros

**2** ❑ 30 - 64 euros

**3** ❑ 65 - 100 euros

**4** ❑ More than 100 euros

1. **QUESTIONS ABOUT THE VISIT**
2. How many people are with you on this trip?

**1** ❑ 1 person

**2**  ❑ 2 to 4 people

**3** ❑ More than 4 people

1. Has the PDO or gastronomic route met your expectations?

**1**  ❑ Sí

**2** ❑ No

1. What would you improve?

**1** ❑ Nothing

**2** ❑ Signage

**3**  ❑ Explanation of the route or the PDO

**4** ❑ More audio-visual media

**5** ❑ Other

1. ¿ Would you be interested in receiving more information after the visit?

**1** ❑ Yes, if it is free of charge

**2**  ❑ Yes, in any case

**3**  ❑ I do not consider it necessary

1. Did you come expressly because of this gastronomic route or did you learn of it while in Andalusia?

**1** ❑ I came expressly because of the route

**2** ❑ I learned about it in Andalusia

1. Does the price paid seem reasonable?

**1** ❑ Sí

**2** ❑ No

1. How did you learn about the route?

**1** ❑ Travel agency

**2** ❑ On the internet, through social networks

**3** ❑ Recommended by friends and family

**4** ❑ Brochures

**5** ❑ Other media

1. Would you try a similar route in the future?

**1** ❑ Sí

**2** ❑ No

1. Were you satisfied with the visit?

**1** ❑ less tan 25%

**2** ❑ 25-50%

**3**  ❑ 51-75%

**4** ❑ 76-99%

**5** ❑ 100%

1. **QUESTIONS ABOUT THE MOTIVATION FOR THE VISIT**
2. What motivated you to visit?

**1** ❑ Learn the culinary tradition of the place

**2** ❑ Learn the process of making wine, oil, ham, etc.

**3**  ❑ Attend food festivals

**4** ❑ Visit ham, oil and wine museums.

1. How do you assess the tourism management at the sites you have visited?

**1** ❑ Good

**2** ❑ Regular

**3**  ❑ Bad

1. What do you think about the creation of a combined route for various gastronomic products and theatrical productions at destinations?

**1** ❑ I agree

**2** ❑ I do not agree; I prefer single gastronomic routes, not combined

1. **EVALUATION OF SERVICES**
2. Evaluate (1 strongly disagree, 10 strongly agree)

|  | ***Assessment(1 strongly disagree, 10 strongly agree)*** | | | | | | | | | |
| --- | --- | --- | --- | --- | --- | --- | --- | --- | --- | --- |
|  | **1** | **2** | **3** | **4** | **5** | **6** | **7** | **8** | **9** | **10** |
| ACCOMODATION |  |  |  |  |  |  |  |  |  |  |
| CATERING |  |  |  |  |  |  |  |  |  |  |
| LEISURE-ENTERTAINMENT |  |  |  |  |  |  |  |  |  |  |
| TRANSPORTATION |  |  |  |  |  |  |  |  |  |  |
| QUALITY OF THE SERVICE |  |  |  |  |  |  |  |  |  |  |
| PUBLIC SAFETY |  |  |  |  |  |  |  |  |  |  |
| HEALTH CARE |  |  |  |  |  |  |  |  |  |  |
| CARE AND TREATMENT |  |  |  |  |  |  |  |  |  |  |
| quality / price ratio |  |  |  |  |  |  |  |  |  |  |
| COMMUNICATION NETWORK |  |  |  |  |  |  |  |  |  |  |
| CLEANLINESS |  |  |  |  |  |  |  |  |  |  |
| TOURISTIC SIGNAGE |  |  |  |  |  |  |  |  |  |  |
| TOURISTIC INFORMATION |  |  |  |  |  |  |  |  |  |  |
| CULTURAL HERITAGE |  |  |  |  |  |  |  |  |  |  |
| SYNTHETIC INDEX OF... |  |  |  |  |  |  |  |  |  |  |

Thank you for your participation


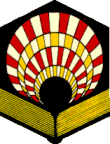
[
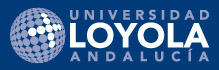
](http://www.uloyola.es/)

CUESTIONARIO PARA REALIZAR UN ESTUDIO SOBRE EL PERFIL DEL TURISTA DE TURISMO GASTRONÓMICO EN ANDALUCÍA

Encuesta para el análisis de la oferta y la demanda de Turismo Activo en Córdoba

**Protocolo**

Dentro del proceso de investigación que estamos llevando a cabo sobre el estudio de la realidad social del turismo gastronómico en Andalucía. Pedimos su valiosa colaboración con el objeto de establecer un estudio científico y riguroso sobre la demanda de turismo gastronómico en Andalucía.

Agradecemos su colaboración en este ambicioso proyecto que pretende dar a conocer, propagar y ayudar a difundir la gastronomía. Rogamos que trate de responder lo más fielmente posible a nuestro cuestionario, sin la necesidad de sentirse coaccionado por pregunta alguna y con la plena libertad de contestar.

Finalmente, le queremos asegurar que los datos obtenidos en este cuestionario serán tratados siempre de forma confidencial, una vez analizados y valorados efectuaremos una memoria que será entregada la Delegación Turismo de córdoba, para que lo haga llegar a todas las empresas del sector que hayan participado en esta encuesta, o en su defecto serán publicadas en una revista de ámbito internacional.

Muchas gracias por su participación

1. **CARACTERÍSTICAS PERSONALES DEL TURISTA GASTRONÓMICO**
2. Sexo:

**1** ❑ Hombre

**2** ❑ Mujer

1. Edad:

**1** ❑ Menor de 18 años

**2** ❑ 18-29 años

**3** ❑ 30-39 años

**4** ❑ 40-49 años

**5** ❑ 50-59 años

**6** ❑ 60 años y más

1. ¿Cuál es su nivel de estudios?

**1** ❑ Sin estudios terminados

**2** ❑ Estudios primarios

**3** ❑ Estudios secundarios

**4** ❑ Estudios superiores

1. ¿Cuál es su estado civil?

**1** ❑ Soltero/a

**2** ❑ Casado/a

**3** ❑ Divorciado/a separado/a

**4** ❑ Otros estados

1. ¿Con qué tipo de renta mensual se identificaría su unidad familiar (euros al mes)?

**1** ❑ Alta (más de 2.500 euros)

**2** ❑ Media-alta (de 2.001 a 2.500 euros)

**3** ❑ Media-media (de 1.501 a 2.000 euros)

**4** ❑ Media-baja (de 1.001 a 1.500 euros)

**5** ❑ Baja (menos de 1.000 euros al mes)

1. ¿Con quién ha realizado la ruta?

**1** ❑ Solo/a

**2** ❑ Acompañado de mi pareja

**3** ❑ Con familiares

**4**  ❑ Con amigos

1. ¿Cuál es su procedencia?

**1** ❑ Andalucía

**2** ❑ Resto de España (Excepto Andalucía)

**3** ❑ Unión Europea (Excepto España)

**4**  ❑ Resto del mundo (Excepto Unión Europea)

1. ¿Cuál es su situación laboral?

**1** ❑ Empleado por cuenta ajena

**2** ❑ Empleado por cuenta propia

**3**  ❑ Jubilado

**4** ❑ Desempleado

**5**  ❑ Estudiante

1. Duración del viaje

**1** ❑ Menos de 24 horas

**2**  ❑ De 2 a 3 días

**3**  ❑ Más de 3 días

1. ¿Cuál ha sido su gasto diario en este viaje?

**1** ❑ Menos de 30 euros

**2** ❑ De 30 a 64 euros

**3** ❑ De 65 a 100 euros

**4** ❑ Más de 100 euros

1. **PREGUNTAS SOBRE LA VISITA REALIZADA**
2. Número de personas que han venido con usted a realizar la ruta:

**1** ❑ 1 persona

**2**  ❑ De 2 a 4 personas

**3** ❑ Más de 4 personas

1. ¿La DOP o la ruta gastronómica ha cubierto sus expectativas respecto a la ruta de turismo gastronómico?

**1**  ❑ Sí

**2** ❑ No

1. ¿Qué mejoraría?

**1** ❑ Nada

**2** ❑ Señalética

**3**  ❑ Explicación de la ruta o de la DOP

**4** ❑ Más medios audiovisuales

**5** ❑ Otros.

1. ¿Estaría interesado en recibir más información después de la visita?

**1** ❑ Sí, si es de forma gratuita

**2**  ❑ Sí, en cualquier caso

**3**  ❑ No lo estimo necesario

1. ¿Vino expresamente para realizar esta ruta gastronómica, o estando en Andalucía se la ofrecieron?

**1** ❑ Vine expresamente de mi lugar de origen

**2** ❑ Fue circunstancial, me la ofrecieron

1. ¿El precio pagado le parece acorde a la ruta?

**1** ❑ Sí

**2** ❑ No

1. ¿Cómo conoció la ruta?

**1** ❑ Agencia de viajes

**2** ❑ Por Internet, mediante redes sociales

**3** ❑ Por recomendación de amigos y familiares

**4** ❑ Por folletos impresos

1. Repetiría experiencia con una ruta similar

**1** ❑ Sí

**2** ❑ No

1. Grado de satisfacción de la visita realizada

**1** ❑ -25%

**2** ❑ 25-50%

**3**  ❑ 51-75%

**4** ❑ 76-99%

**5** ❑ 100%

1. **PREGUNTAS SOBRE LA MOTIVACIÓN DE LA VISITA**
2. ¿Qué le motiva más de la visita?

**1** ❑ Conocer la tradición culinaria del lugar

**2** ❑ Conocer el proceso de elaboración del vino, aceite, jamón.

**3**  ❑ Asistir a festivales gastronómicos

**4** ❑ Visitar museos del jamón, aceite, vino,etc

1. ¿Cómo valora usted la situación actual en cuanto a la gestión turística de sitios como los que ha visitado?

**1** ❑ Buena

**2** ❑ Regular

**3**  ❑ Mala

1. ¿Qué opina sobre la creación de una ruta combinada de varios productos gastronómicos con representación teatral?

**1** ❑ Estoy de acuerdo

**2** ❑ No estoy de acuerdo, prefiero visitar una sola ruta gastronómica

1. **VALORACIONES Y OPINIONES**
2. Por favor, valores del 1 al 10, siendo 1 totalmente en desacuerdo y 10 totalmente de acuerdo, a cada uno de los siguientes aspectos relacionados con esta zona como potencial destino turístico:

| ***Ítems a valorar*** | ***Valoraciones*** | | | | | | | | | | | | | | | | | | | | | | | | | | | | | |
| --- | --- | --- | --- | --- | --- | --- | --- | --- | --- | --- | --- | --- | --- | --- | --- | --- | --- | --- | --- | --- | --- | --- | --- | --- | --- | --- | --- | --- | --- | --- |
|  | **1** | | | **2** | | | **3** | | | **4** | | | **5** | | | **6** | | | **7** | | | **8** | | | **9** | | | **10** | | |
| **Alojamiento** |  | | |  | | |  | | |  | | |  | | |  | | |  | | |  | | |  | | |  | | |
| **Restauración** |  | | |  | | |  | | |  | | |  | | |  | | |  | | |  | | |  | | |  | | |
| **Ocio/Diversión** |  | | |  | | |  | | |  | | |  | | |  | | |  | | |  | | |  | | |  | | |
| **Transportes públicos: alquiler de coches** |  | | |  | | |  | | |  | | |  | | |  | | |  | | |  | | |  | | |  | | |
| **Calidad de la Oferta turística: Paisajes y parques** | | |  | | |  | | |  | | |  | | |  | | |  | | |  | | |  | | |  | | |  |
| **Calidad de la Oferta turística: Entornos urbanos** | |  | | |  | | |  | | |  | | |  | | |  | | |  | | |  | | |  | | |  | |
| **Seguridad ciudadana** |  | | |  | | |  | | |  | | |  | | |  | | |  | | |  | | |  | | |  | | |
| **Asistencia sanitaria** |  | | |  | | |  | | |  | | |  | | |  | | |  | | |  | | |  | | |  | | |
| **Atención/Trato** |  | | |  | | |  | | |  | | |  | | |  | | |  | | |  | | |  | | |  | | |
| **Relación Precio/Servicio** |  | | |  | | |  | | |  | | |  | | |  | | |  | | |  | | |  | | |  | | |
| **Red de comunicaciones** |  | | |  | | |  | | |  | | |  | | |  | | |  | | |  | | |  | | |  | | |
| **Limpieza** |  | | |  | | |  | | |  | | |  | | |  | | |  | | |  | | |  | | |  | | |
| **Señalización turística** |  | | |  | | |  | | |  | | |  | | |  | | |  | | |  | | |  | | |  | | |
| **Información turística** |  | | |  | | |  | | |  | | |  | | |  | | |  | | |  | | |  | | |  | | |
| **Patrimonio cultural** |  | | |  | | |  | | |  | | |  | | |  | | |  | | |  | | |  | | |  | | |
| **Índice sintético de percepción** |  | | |  | | |  | | |  | | |  | | |  | | |  | | |  | | |  | | |  | | |

Muchas gracias por su colaboración
