# Supplementary material for: Analysis of the demand for gastronomic tourism in Andalusia (Spain)
Source: PLoS One. 2021 Feb 5;16(2):e0246377. doi: 10.1371/journal.pone.0246377 (PMC7864416; doi:10.1371/journal.pone.0246377)
Supplement: Attachment — Submitted filename: carta a revisores.docx [file pone.0246377.s003.docx]

Dear editor:

We are grateful to *Plos One* for giving us the opportunity to publish our article “Analysis of the demand for gastronomic tourism in Andalusia (Spain)” .

We have made the changes to the manuscript to address the issues reviewers have raised.

We have removed the figures from within the manuscript file.

We have provide additional details regarding participants consent, in the setion methodos.

The authors have not received specific funding for this work.

Kind regards,

Genoveva Millán

Editor:

Please ensure that you refer to Figure 2 and 4 in your text as, if accepted, production will need this reference to link the reader to the figure. References to figures 2 and 4 have been included in the text

. We note you have included a table to which you do not refer in the text of your manuscript. Please ensure that you refer to Table 2 and 3 in your text; References to tables 2 and 3 have been included in the text

Reviewers' comments:

We appreciate the reviewer's comments and suggestions and have made them

Reviewer #1: The reviewed article has been well prepared in terms of its content and methodology. The introduction, however long (maybe even too extensive ...), places the reader well in the realities of research. Well-chosen methodology (adequate to the research subject).
I wonder about the literature review - are the author / authors deliberately omitting similar research from European countries? I am thinking in particular of countries with a fragmented structure of farms, for which culinary tourism and culinary heritage are an important alternative to the development and maintenance of the character of rural areas - Italy, Romania, Lithuania, Poland. There are quite a few studies from these areas. The references indicated by the reviewer have been included in the text.

Reviewer #2:

Dear Reviewer:

We appreciate the reviewer's comments and suggestions and have made them

The article presented deals with a very interesting and important topic from the economic point of view.
The article is well structured, although I recommend the following changes:

- Autonomous Community, as such, does not exist in English. I recommend using the word "region".

The word autonomous community has been changed to region.

- I think it is convenient to separate the discussion section from the conclusions of the work, since they are totally different aspects.

The discussion has been separated from the conclusions

- Reinforce the literature and bibliography in English. New bibliography has been introduced in English
